# Is Physiologic Stress Test with Imaging Comparable to Anatomic Examination of Coronary Arteries by Coronary Computed Tomography Angiography to Investigate Coronary Artery Disease? – A Systematic Review and Meta-Analysis

**DOI:** 10.7759/cureus.6941

**Published:** 2020-02-10

**Authors:** Waqas J Siddiqui, Muhammad Shabbir Rawala, Waqas Abid, Muhammad Zain, Murrium I Sadaf, Danish Abbasi, Chikezie Alvarez, Farah Mansoor, Syed Farhan Hasni, Sandeep Aggarwal

**Affiliations:** 1 Cardiology/Nephrology, Drexel University College of Medicine, Philadelphia, USA; 2 Internal Medicine, Charleston Area Medical Center, Charleston, USA; 3 Interventional Radiology, Christiana Hospital, Newark, USA; 4 Internal Medicine, Sheikh Zayed Medical College and Hospital, Rahim Yar Khan, PAK; 5 Internal Medicine, Yale School of Medicine, New Haven, USA; 6 Cardiovascular Diseases, University of Arkansas, Little Rock, USA; 7 Medicine, St. Francis Medical Center, Trenton, USA; 8 Medicine, Dow University, Karachi, PAK; 9 Heart Failure and Transplant, Albert Einstein Hospital, Philadelphia, USA; 10 Nephrology, University of Pennsylvania, Philadelphia, USA

**Keywords:** angina, computed tomography angiography, cardiac imaging, coronary cta

## Abstract

Objective

Coronary computed tomography angiography (CCTA) is a noninvasive diagnostic modality that remains underutilized compared to functional stress testing (ST) for investigating coronary artery disease (CAD). Several patients are misdiagnosed with noncardiac chest pain (CP) that eventually die from a cardiovascular event in subsequent years. We compared CCTA to ST to investigate CP.

Methods

We searched MEDLINE, PubMed, Cochrane Library, and Embase from January 1, 2007 to July 1, 2018 for randomized controlled trials (RCTs) comparing CCTA to ST in patients who presented with acute or stable CP. We used Review Manager (RevMan) [Computer program] Version 5.3 (Copenhagen: The Nordic Cochrane Centre, The Cochrane Collaboration, 2014) for review and analysis.

Results

We included 16 RCTs enrolling 21,210 patients; there were more patients with hyperlipidemia and older patients in the ST arm compared to the CCTA arm. There was no difference in mortality: 103 in the CCTA arm vs. 110 in the ST arm (risk ratio [RR] = 0.93, 95% confidence interval [CI] = 0.71-1.21, P = .58, and I^2^ = 0%). A significant reduction was seen in myocardial infarctions (MIs) after CCTA compared to ST: 115 vs. 156 (RR = 0.71, CI = 0.56-0.91, P < .006, I^2^=0%). On subgroup analysis, the CCTA arm had fewer MIs vs. the ST with imaging subgroup (RR = 0.70, CI = 0.54-0.89, P = .004, I^2 ^= 0%) and stable CP subgroup (RR = 0.66, CI = 0.50-0.88, P = .004, I^2 ^= 0%). The CCTA arm showed significantly higher invasive coronary angiograms and revascularizations and significantly reduced follow-up testing and recurrent hospital visits. A trend towards increased unstable anginas was seen in the CCTA arm.

Conclusions

Our analysis showed a significant reduction in downstream MIs, hospital visits, and follow-up testing when CCTA is used to investigate CAD with no difference in mortality.

## Introduction

Coronary heart disease is one of the leading causes of death, globally. Annually, more than 20 million patients undergo workup for angina [1]. Patients misdiagnosed with noncardiac chest pain (CP) have died from a cardiovascular event five years from the misdiagnosis [2]. Therefore, it is essential to identify patients at the highest risk of coronary artery disease (CAD) who may benefit from a workup using invasive coronary angiography (ICA) and subsequent revascularization. Coronary computed tomography angiography (CCTA) is 89% sensitive and 96% specific for the diagnosis of CAD, and CCTA is becoming an alternative to ICA due to its comparatively high diagnostic accuracy and noninvasive approach [3-5]. In fact, current cardiology guidelines recommend using CCTA to diagnose CAD [6].

## Materials and methods

We conducted a systematic review and meta-analysis to compare CCTA to ST with subgroup analyses of ST (with and without imaging which has never been done before) and CP (acute chest pain [ACP] or stable chest pain [SCP]). Over the years, few meta-analyses comparing CCTA to ST have been published, and the outcomes are variable; these are summarized in Table 1 [7-11].

**Table 1:** Characteristics of previously published meta-analyses ACS, acute coronary syndrome; CAD, coronary artery disease; CCTA, coronary computed tomography angiography; CP, chest pain; ED, emergency department; FST, functional stress testing; ICA, invasive coronary angiography; MI, myocardial infarction; UC, usual care.

Meta-analysis	Studies (n)	Participants (n)	Results	Conclusion
D'Ascenzo et al. 2013 [7]	4	2,567	Patients in the CCTA group were more likely to undergo coronary revascularization in the future. Time to diagnosis was reduced along with the reduced cost of care in the ED.	CCTA proved to be cost-effective in limited data along with a higher number of invasive coronary revascularization procedures.
Hulten et al. 2013 [8]	4	3,266	CCTA did not show any mortality benefits, increased incidence of MI, and or rehospitalization after ED discharge. However, CCTA decreased the length of ED stay and ED cost. CCTA was associated with increased ICA and coronary revascularization.	The use of CCTA decreased the length of ED stay as well as ED cost but increased the incidence of ICA and revascularization.
El-Hayek et al. 2014 [9]	7	6,058	CCTA reduced the risk of ACS and repeat ED visits in the future but with higher rates of revascularization procedures. There was no difference in ICA.	CCTA use in the ED for patients with low to intermediate risk of CAD reduces the risk of future ACS and subsequent ED visits for CP.
Bittencourt et al. 2016 [10]	4	14,817	Compared to UC, the CCTA showed a reduced annual rate for MI and cardiac CP but no difference in all-cause mortality. A higher rate of ICA and revascularization were also seen among patients undergoing CCTA.	Although CCTA reduced the rate of MI, it increased the rate of ICA and revascularization in patients with stable CAD.
Foy et al. 2017 [11]	13	20,092	Compared to FST, CCTA showed reduced incidence of MI but a higher incidence of ICA and revascularization. CCTA use also increased the number of new CAD diagnosis and new prescription of aspirin and statins. However, despite all this, no mortality difference was noted between CCTA and FST.	CCTA increases the incidence of new CAD diagnosis with a higher number of invasive coronary angiography and revascularization but reduces the risk of MI in the future.

Data sources and searches

We completed a systematic review according to the Preferred Reporting Items for Systematic Review and Meta-analyses (PRISMA) guidelines [12]. We searched MEDLINE, PubMed, Cochrane Library, and Embase from January 1, 2007 to July 1, 2018 for RCTs, comparing CCTA to ST for suspected underlying CAD in patients who presented with CP. We combined search terms using the Boolean operator OR. Our search strategy included (Coronary Computed Tomography Angiography) OR (CCTA) OR (Coronary CTA) OR (Coronary CT Angiography). Due to the advancement in multislice CT technology, we only included studies performed after 2007. After duplicates were removed, a total of 405 studies were identified.

Study selection

**Figure 1:**
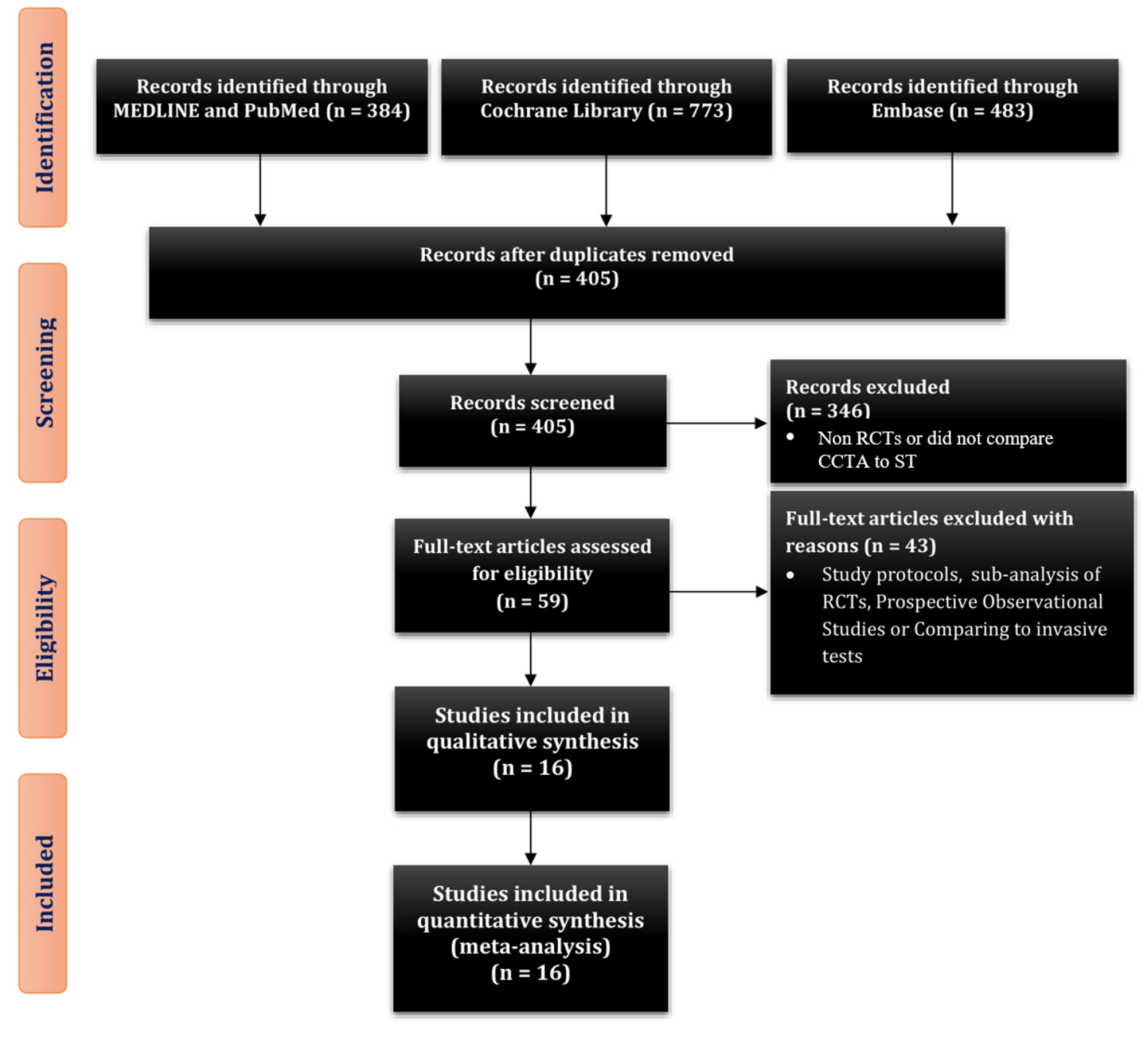
PRISMA 2009 study flow diagram PRISMA, preferred reporting items for systematic reviews and meta-analyses; RCT, randomized control trial; CCTA, coronary computed tomography angiography; ST, stress testing.

Three reviewers (W.J.S., W.A., and M.S.R.) reviewed the abstracts and selected 59 articles for a full review. A total of 16 RCTs met the predefined inclusion criteria for qualitative and quantitative analysis comparing CCTA to ST: myocardial perfusion imaging or scan, stress electrocardiogram (bicycle or treadmill), stress echocardiogram, pharmacologic nuclear scan, graded exercise testing, and pharmacologic ST (Figure 1) [13-28].

Inclusion criteria

We used the following inclusion criteria: prospective RCTs, RCTs comparing CCTA to ST after CP, age ≥ 18 years, study population ≥ 50 patients, and follow-up ≥ four weeks.

Data extraction and quality assessment

W.J.S., M.S.R., and W.A. extracted data into predefined fields on a Microsoft Excel sheet for baseline characteristics and study outcomes. W.J.S. cross-checked the data and made the necessary corrections. All three reviewers discussed the revisions and agreed to the final entry.

Data synthesis and analysis

Statistical Method

**Figure 2:**
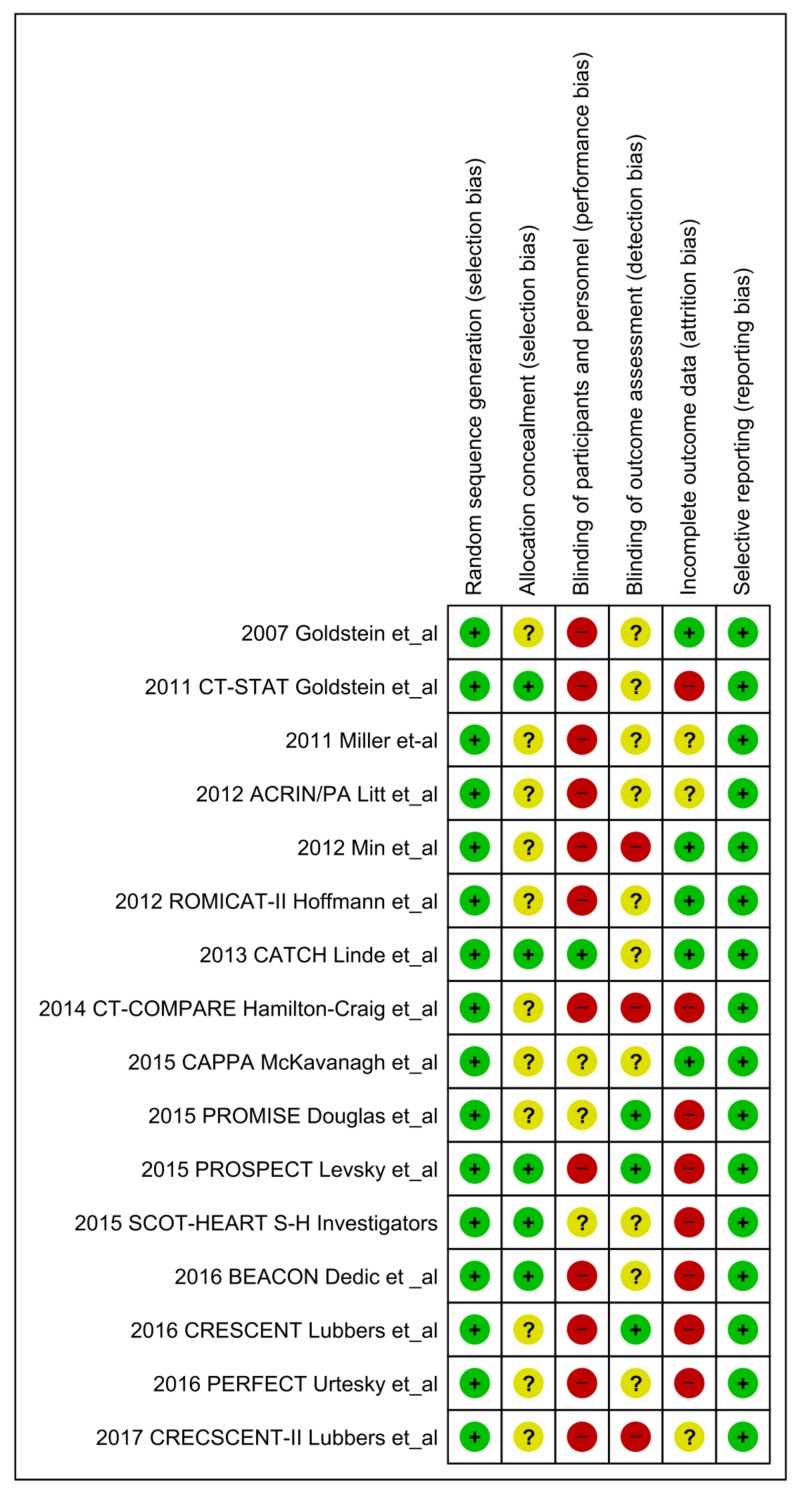
Cochrane Collaboration’s tool for the quality assessment of randomized controlled trials Bias risk presented in 16 studies [13-28]

We used a random-effects model and Mantel-Haenszel method for dichotomous data to calculate the relative risk (RR) and odds ratio (OR), and inverse variance for the continuous data to estimate the standardized mean difference in Review Manager (RevMan) [Computer program] Version 5.3 (Copenhagen: The Nordic Cochrane Centre, The Cochrane Collaboration, 2014). We reported results as forest plots. We used online GraphPad Online Version 8 (GraphPad Software, La Jolla, California, USA) to compare the baseline characteristics and to calculate the number needed to treat (NNT) to prevent one adverse event. A two-tailed P-value of < .05 was considered statistically significant. We assumed a 1:1 ratio in each arm except for the ACRIN/PA trial, which randomized patients in a 2:1 ratio where we used the same ratio for outcomes; this failed to uncover any event in both arms [19]. Baseline characteristics are summarized in Table 2 and Table 3 [13-28]. The salient features of each RCT are outlined in Table 4 [13-28]. We used the Cochrane Collaboration’s tool for the quality assessment of RCTs (Figure 2, Table 5) [13-28].

**Table 2:** Baseline characteristics BMI, body mass index; CCTA, coronary computed tomography angiography; DM, diabetes mellitus; HLD, hyperlipidemia; HTN, hypertension; N/A, not applicable; ST, stress testing.

	Intervention	n	Age	Male %	Female %	BMI (kg/m^2^)	HTN %	HLD %	DM %	Smoker %	Aspirin
Goldstein et al. 2007 [15]	CCTA	99	48±11	43	57	29±5	39	34	8.2	15	24
	ST	98	51 ±12	57	43	29±5	38	38	12.2	20	29
CT-STAT Goldstein et al. 2011 [16]	CCTA	361	50±10	45.2	54.8	28.1±4.7	35.5	31	5.5	25.2	24.9
	ST	338	50±10	47	53	28.7±5.1	38.8	36.1	8.3	19.5	30.5
Miller et-al. 2011 [17]	CCTA	30	51±10	43	57	N/A	N/A	N/A	N/A	N/A	N/A
	ST	30	51±10	57	43	N/A	N/A	N/A	N/A	N/A	N/A
ACRIN Litt et al. 2012 [19]	CCTA	908	49±9	49	51	N/A	51	27	14	32	22
	ST	462	50±10	44	56	N/A	50	26	14	34	25
Min et al. 2012 [20]	CCTA	91	55.9±10	58	42	N/A	62	53	23	58	N/A
	ST	89	58.9±9.5	43	57	N/A	59	61	21	44	N/A
ROMICAT-II Hoffmann et al. 2012 [18]	CCTA	501	54±8	52	48	29.4±5.3	54	46	17	50	23
	ST	499	54±8	54	46	29.1±4.8	54	45	17	49	23
CATCH Linde et al. 2013 [21]	CCTA	285	56.4±12.2	56.5	43.5	28	47.4	41.1	12.3	60.4	N/A
	ST	291	54.9±12.2	57.7	42.3	28	36.4	34.7	10	67	N/A
CT-COMPARE Hamilton-Craig et al. 2014 [22]	CCTA	322	52.2±10.7	59	41	N/A	31	25	7	24	N/A
	ST	240	52.3±9.8	58	42	N/A	31	24	6	23	N/A
CAPPA McKavanagh et al. 2015 [26]	CCTA	243	57.8±10.0	56.8	43.2	27.8±3.6	31.7	N/A	5.8	19%	N/A
	ST	245	58.9±10.2	53.5	46.5	28±3.6	29.8	N/A	4.9	19	N/A
PROMISE Douglas et al. 2015 [24]	CCTA	4996	60.7±8.3	48.1	51.9	30.5±6.1	65	67.4	21.3	50.7	45.2
	ST	5007	60.9±8.3	46.6	53.4	30.5±6.1	65	67.9	21.5	51.4	44.2
PROSPECT Levsky et al. 2015 [25]	CCTA	200	56.8±11.8	37	63	30.5±6.2	70.5	49	33	17	39
	ST	200	56.3±10.5	37.5	62.5	30.7±6.6	73.5	55	31	13	36
SCOT-HEART S-H Investigators 2015 [23]	CCTA	2073	57.1±9.7	N/A	N/A	29.7±5.8	34	53	11	53	49
	ST	2073	57.0±9.7	N/A	N/A	29.8±6	33	52	11	53	48
CRESCENT Lubbers et al. 2016 [14]	CCTA	242	55±10	45	55	28±5	52	54	17	34	29
	ST	108	55±10	44	56	28±5	52	61	16	36	29
BEACON Dedic et al. 2016 [27]	CCTA	250	55±10	51	49	N/A	36	43	12	47	19
	ST	250	53±9	55	45	N/A	35	45	13	40	14
PERFECT Uretsky et al. 2016 [28]	CCTA	206	59 ±10	46	54	N/A	68	43	24	45	40
	ST	205	60 ±10	47	53	N/A	69	53	33	46	44
CRECSCENT-II Lubbers et al. 2018 [13]	CCTA	130	58±11	51	49	28±5	52	38	18	33	N/A
	ST	138	58±11	44	56	28±5	52	40	18	42	N/A

**Table 3:** Comparing baseline characteristics BMI, body mass index; CCTA, coronary computed tomography angiography; DM, diabetes mellitus; HLD, hyperlipidemia; HTN, hypertension; N/A, not applicable; ST, stress testing.

Intervention	CCTA	ST	Mean Difference	95% Confidence Interval	P-value
n	10,937	10,273			
Age	57.4±10	58±9.8	-0.600	-0.867 to -0.333	< .001
BMI (kg/m^2^)	30±5.9 (8,845)	30.1±5.9 (8706)	-0.1	-0.275 to 0.075	.26
Male % (n/total)	49.4 (4,379/8,864)	49.7 (4,075/8,200)	N/A	N/A	.71
Female % (n/total)	50.6 (4485/8864)	50.3 (4,125/8,200)	N/A	N/A	.71
HTN % (n/total)	48.6 (5301/10,907)	47.8 (4,896/10,243)	N/A	N/A	.2482
HLD % (n/total)	43.2 (4,607/10,664)	45.6 (4,559/9,998)	N/A	N/A	.0006
DM % (n/total)	15.3 (1,669/10,907)	15.8 (1,618/10,243)	N/A	N/A	.3310
Smoker % (n/total)	37.5 (4,090/10,907)	37.1 (3,800/10,243)	N/A	N/A	.9070
Aspirin % (n/total)	31.5 (3,127/9,927)	32.3 (3013/9,329)	N/A	N/A	.2417

**Table 4:** Characteristics of randomized control trials ~ Traditional Care = Graded exercise testing/Pharmacologic stress testing * Stress Test = Stress Echocardiography/MPI # Functional testing = Exercise ECG, Exercise or Pharmacologic Nuclear Stress Testing, and Stress Echocardiography Ѱ SOC = Standard Optimal Care CCT, cardiac computerized tomography; CCTA, coronary computed tomography angiography; ECG, electrocardiography; EST, exercise stress electrocardiography test; F/u, follow up; JACC, Journal of American College of Cardiology; JCCT, Journal of Cardiovascular Computed Tomography; MPI, myocardial perfusion imaging; MPS, myocardial perfusion scan; MSCT, multi-slice computed tomographic angiography; NEJM, New England Journal of Medicine; NSTE-ACS, non-ST elevated acute coronary syndrome; RCT, randomized control trial; SC, standard care; SE, standard evaluation; SOC, standard of care; w/, with.

Name	Design	Country	Publication Year	Journal	Enrollment	Population	Setting	Intervention vs Comparison	F/u Duration	CT Scanners
Goldstein et al. 2007 [15]	RCT	United States	2007	JACC	March 2005 – September 2005	Acute chest pain	Emergency Department	MSCT vs rest-stress MPI	6 months	64-slice MSCT scanner (Sensation 64 Cardiac, Siemens Medical Systems, Forchheim, Germany)
CT-STAT Goldstein et al. 2011 [16]	Multicenter, comparative effectiveness RCT	United States	2011	JACC	June 2007 –November 2008	Acute Chest pain	Emergency Department	CCTA vs rest-stress MPI	6 months	64-slice MSCT scanner (Sensation 64 Cardiac, Siemens Medical Systems, Forchheim, Germany)
Miller et al. 2011 [17]	Single-center RCT	United States	2011	Academic Emergency Medicine	October 20, 2008 – February 02, 2009	Acute chest pain	Emergency Department	SC+CCTA vs SC	3 months	64-slice multidetector CT scanner (Toshiba America Medical Systems, Inc., Tustin, CA)
ACRIN/PA Litt et al. 2012 [19]	Multicenter RCT	United States	2012	NEJM	July 07, 2009 –November 03, 2011	Acute Chest pain	Emergency Department	CCTA vs Traditional care^~^	1 month	64-slice or greater multidetector CT scanner
Min et al. 2012 [20]	Multicenter (2 centers) RCT	United States	2012	JCCT	December 2008 – June 2009	Stable chest pain	Outpatient	CCTA vs. MPS	2 months	64-detector row CT scanner (Lightspeed VCT; GE Healthcare, Milwaukee, WI)
ROMICAT-II Hoffmann et al. 2012 [18]	Multicenter RCT	United States	2012	NEJM	April 23, 2010 –January 30, 2012	Acute chest pain	Emergency Department	CCTA vs. SE	28 Days	64-slice CT technology
CATCH Linde et al. 2013 [21]	Single-center RCT	Denmark	2013	International Journal of Cardiology	January 2010 –January 2013	Acute chest pain	Hospitalized w/ suspicion of NSTE-ACS, d/c within 24 hours	CCTA vs. Bicycle exercise-ECG and/or MPI	4 months	320 multidetector scanner (Aquilion One, Toshiba Medical systems)
CT-COMPARE Hamilton-Craig et al. 2014 [22]	Single-center RCT	Australia	2014	International Journal of Cardiology	March 2010 –April 2011	Acute chest pain	Emergency Department	CCTA vs Exercise ECG	12 months	(Somaton Definition 64 detector, or Definition Flash 128-detector; Siemens, Erlangen, Germany)
CAPPA McKavanagh et al. 2015 [26]	Single-center RCT	Ireland	2015	European Heart Journal	September 2010 – November 2011	Stable chest pain	Outpatient	CCT vs. EST	12 months	64-detector platform (Philips Brilliance 64 Cleveland, Ohio, USA)
PROMISE Douglas et al. 2015 [24]	Multicenter, comparative effectiveness RCT	United States	2015	NEJM	July 27, 2010 – September 19, 2013	Stable chest pain	Outpatient	CCTA vs. Functional testing^#^	25 months	64-slice or greater multidetector CT scanner
PROSPECT Levsky et al. 2015 [25]	Single-center, comparative effectiveness RCT	United States	2015	Annals of Internal Medicine	July 2008 – March 2012	Acute chest pain	Telemetry Inpatient Ward	CCTA vs. MPI	12 months	64 –detector-row scanners
SCOT-HEART S-H Investigators 2015 [23]	Open-label, parallel-group Multicenter RCT	Scotland	2015	Lancet	November 18, 2010 – September 24, 2014	Stable chest pain	Outpatient	CCTA + SOC vs SOC	20 months (1.7 Years)	64-row scanners (Brilliance 64, Philips Medical Systems, Biograph mCT Siemens) and 320 detector row scanners (Aquilion ONE, Toshiba Medical Systems)
CRESCENT Lubbers et al. 2016 [14]	Multicenter RCT	Netherland	2016	European Heart Journal	April 2011 – July 2013	Stable chest pain	Outpatient	CCT vs. Functional testing	12 months	64-slice or more advanced CT technology, with radiation minimizing measures
BEACON Dedic et al. 2016 [27]	Multicenter, Prospective, open-label, RCT	Netherland	2016	JACC	July 11, 2011 - January 30, 2014	Acute chest pain	Emergency Department	CCTA vs. SOC^Ѱ^	30 days	64-slice or more advanced CT technology, using ECG-synchronized axial or spiral scan protocols
PERFECT Uretsky et al. 2016 [28]	Single-center, comparative effectiveness RCT	United States	2016	Journal of Nuclear Cardiology	July 2011 – December 2013	Acute chest pain	Inpatient	CCTA vs. Stress Test ^*^	12 months	(Toshiba Aquilion 64-detector Toshiba America Medical Systems, Tustin, CA, or Siemens Somatoform Sensation 64-detector, Siemens Medical Solutions USA, Malvern, PA).
CRESCENT-II Lubbers et al. 2017 [13]	Multicenter RCT	Netherland	2017	JACC	July 2013 – November 2015	Stable Angina	Outpatient	CCT vs. Functional testing	6 months	Somatom Definition Flash and Force Siemens Healthineers, Forchheim, Germany

**Table 5:** Cochrane risk of bias for quality assessment CTA, computed tomography angiogram.

Name	Random Sequence	Allocation Concealment	Blinding of Participants and Personnel	Blinding of Outcome Assessment	Incomplete Outcome Data	Reporting Bias
Goldstein et al. 2007 [15]	Yes via SAS software version 9.1	Not reported	No	Not reported	No	Low risk
	Low risk	Unclear	High risk	Unclear	Low risk	
CT-STAT Goldstein et al. 2011 [16]	1:1 ratio, alternating block design	Randomization envelopes	No	Not reported	Yes	Low risk
	Low risk	Low risk	High risk	Unclear	High risk	
Miller et al. 2011 [17]	1:1 ratio in an open-label fashion	Not reported	No	Not reported	Not reported	Low risk
	Low risk	Unclear	High risk	Unclear	Unclear	
ACRIN/PA Litt et al. 2012 [19]	Computer-based randomization, 2:1 ratio	Not reported	No	Not reported	Not reported	Low risk
	Low risk	Unclear	High Risk	Unclear	Unclear	
Min et al. 2012 [20]	1:1 ratio, simple randomization stratified by site	Not reported	No	No	No	Low Risk
	Low risk	Unclear	High risk	High risk	Low risk	
ROMICAT-II Hoffmann et al. 2012 [18]	1:1 ratio in the emergency department	Not reported	No	Not reported	No	Low risk
	Low risk	Unclear	High risk	Unclear	Low risk	
CATCH Linde et al. 2013 [21]	Computer-based block randomization, in a 1:1 ratio	Yes	Yes until tests were performed	Not reported	No	Low risk
	Low risk	Low risk	Low risk	Unclear	Low risk	
CT-COMPARE Hamilton-Craig et al. 2014 [22]	Computer-generated random sequence	Not reported	No	No	Yes	Low risk
	Low risk	Unclear	High risk	High risk	High risk	
CAPPA McKavanagh et al. 2015 [26]	Permuted block randomization at the clinic	Not reported	Not reported	Not reported	No	Low risk
	Low risk	Unclear	Unclear	Unclear	Low risk	
PROMISE Douglas et al. 2015 [24]	Yes	Not reported	Not reported	Independent clinical-events committee	Yes	Low risk
	Low risk	Unclear	Unclear	Low risk	High risk	
PROSPECT Levsky et al. 2015 [25]	SAS software-generated, blocked, 1:1 randomization	Sequentially numbered, sealed, opaque envelopes	No	Yes	Yes	Low risk
	Low risk	Low risk	High risk	Low risk	High risk	
SCOT-HEART S-H Investigators 2015 [23]	Web-based randomization in a 1:1 ratio	Yes	Not reported	Not reported	Yes	Low risk
	Low risk	Low risk	Unclear	Unclear	High risk	
CRESCENT Lubbers et al. 2016 [14]	Randomization in 2:1 ratio to CTA or functional testing	Not reported	No	Yes	Yes	Low risk
	Low Risk	Unclear	High risk	Low risk	High risk	
BEACON Dedic et al. 2016 [27]	1:1 computer-generated block randomization	Sealed, sequentially numbered, opaque envelopes	No	Not reported	Yes	Low risk
	Low risk	Low risk	High risk	Unclear	High risk	
PERFECT Uretsky et al. 2016 [28]	Method of randomization not reported	Not reported	No	Not reported	Yes	Low risk
	Low risk	Unclear	High risk	Unclear	High risk	
CRESCENT-II Lubbers et al. 2017 [13]	Method of randomization not reported	Not reported	No	No	Not reported	Low risk
	Low risk	Unclear	High risk	High risk	Unclear	

Heterogeneity

We used I^2^ statistics to calculate the heterogeneity. I^2^ > 50% was considered substantial heterogeneity, as explained in the Cochrane Handbook for Systematic Reviews [29]. We performed a sensitivity analysis for considerable heterogeneity.

## Results

We included 16 RCTs with 21,210 patients (10,937 in the CCTA arm and 10,273 in the ST arm). Patients in the ST arm were older than those in the CCTA arm (57.9 ± 9.8 years vs. 57.4 ± 10 years, respectively; P = .0002) and had more hyperlipidemia (45.62% vs. 43.18%, respectively; P = .0004). There was no difference in baseline body mass index, hypertension, diabetes, smoking status, and baseline use of aspirin. Three studies used ST without imaging for a total of 1,110 patients (595 in the CCTA arm and 515 in the ST without imaging arm) [17,22,26].

Primary endpoints were all-cause mortality and new myocardial infarction (MI) during the follow-up period. Secondary endpoints included ICA after ST, true positive ICA, revascularizations, new unstable anginas, emergency room (ER) visits or hospital admissions during the follow-up period, follow-up tests, complications (stroke, bleeding, anaphylaxis, or renal failure) attributed to CCTA compared to ST, direct discharges from ER, ER cost and total cost, and radiation dose. The results are summarized in Table 6.

**Table 6:** Outcomes * Procedural complications include stroke, bleeding, anaphylaxis, or renal failure Abbreviations: ER, emergency room; ICA, invasive coronary angiography; ST, stress testing.

Outcome	CCTA	ST	Effect Estimate	Confidence Interval	P-value	I^2^
Primary Outcomes						
All-Cause Mortality	103	110	0.93	0.71-1.21	.58	0%
ST with Imaging	100	108	0.92	0.70-1.21	.55	0%
ST without Imaging	3	2	1.26	0.21-7.71	.8	0%
All-Cause Mortality	103	110	0.93	0.71-1.21	.58	0%
Acute Chest Pain	9	12	0.75	0.30-1.89	.54	0%
Stable Chest Pain	103	110	0.95	0.71-1.25	.7	0%
New Myocardial Infarction	115	156	0.71	0.56-0.91	.006	0%
ST with Imaging	108	151	0.7	0.54-0.89	.004	0%
ST without Imaging	7	5	1.14	0.35-3.75	.83	0%
New Myocardial Infarction	115	156	0.71	0.56-0.91	.006	0%
Acute Chest Pain	35	36	0.88	0.54-1.44	.61	0%
Stable Chest Pain	80	20	0.66	0.5-0.88	.004	0%
Secondary Outcomes						
Cumulative ICA	1,044	701	1.41	1.28-1.55	< .00001	1%
ST with Imaging	948	637	1.37	1.21-1.55	< .00001	11%
ST without Imaging	96	64	1.39	1.04-1.85	.02	0%
Cumulative ICA	1,044	701	1.41	1.28-1.55	< .00001	1%
Acute Chest Pain	311	205	1.35	1.13-1.62	.001	8%
Stable Chest Pain	733	496	1.44	1.30-1.61	< .00001	0%
True Positive ICA	629	270	2.85	2.28-3.56	< .00001	0%
ST with Imaging	565	246	2.84	2.25-3.59	< .00001	0%
ST without Imaging	64	24	4.67	1.15-18.91	.03	48%
True Positive ICA	629	270	2.85	2.28-3.56	< .00001	0%
Acute Chest Pain	117	41	3.2	1.83-5.60	< .001	0%
Stable Chest Pain	512	229	2.79	2.19-3.55	< .00001	0%
Cumulative Revascularization	789	472	1.84	1.44-2.35	< .00001	53%
ST with Imaging	737	450	1.77	1.34-2.33	< .0001	60%
ST without Imaging	52	22	2.36	1.40-3.98	.001	0%
Cumulative Revascularization	789	472	1.84	1.44-2.35	< .00001	53%
Acute Chest Pain	175	82	1.95	1.42-2.69	< .0001	17%
Stable Chest Pain	614	390	1.7	1.16-2.51	.007	77%
New Unstable Anginas	257	198	1.18	0.99-1.41	.06	0%
ST with Imaging	245	191	1.18	0.98-1.40	.07	0%
ST without Imaging	12	7	1.09	0.20-5.92	.92	49%
New Unstable Anginas	257	198	1.18	0.99-1.41	.06	0%
Acute Chest Pain	118	84	1.15	0.90-1.48	.27	0%
Stable Chest Pain	139	114	1.21	0.93-1.58	.15	4%
ER visits or hospital admissions	570	616	0.75	0.60-0.94	.01	63%
ST with Imaging	554	551	0.92	0.83-1.02	.11	0%
ST without Imaging	16	65	0.27	0.15-0.48	< .0001	27%
ER visits or hospital admissions	570	616	0.75	0.60-0.94	.01	63%
Acute Chest Pain	300	289	0.86	0.72-1.04	.11	22%
Stable Chest Pain	270	327	0.5	0.21-1.23	.13	86%
Cumulative Follow up Testing	242	342	0.45	0.22-0.90	.02	86%
ST with Imaging	159	197	0.43	0.16-1.14	.09	86%
ST without Imaging	83	145	0.39	0.28-0.56	< .00001	0%
Cumulative Follow up Testing	242	342	0.45	0.22-0.90	.02	86%
Acute Chest Pain	166	165	0.83	0.44-1.55	.56	70%
Stable Chest Pain	76	177	0.17	0.04-0.77	.02	80%
Procedural Complications*	7	7	0.98	0.35-2.74	.96	0%
Direct ER Discharges	936	421	1.45	0.63-3.30	.38	94%
Cost in ER	-	-	-4.68	(-10.38) - (1.01)	.11	100%
Total Downstream Cost	-	-	-0.01	(-0.17) - (0.14)	.85	45%
Cumulative Radiation Dose	7.3±6.6	2.6±6.5	0.47	0.08-0.86	.02	97%

Primary Endpoints

**Figure 3:**
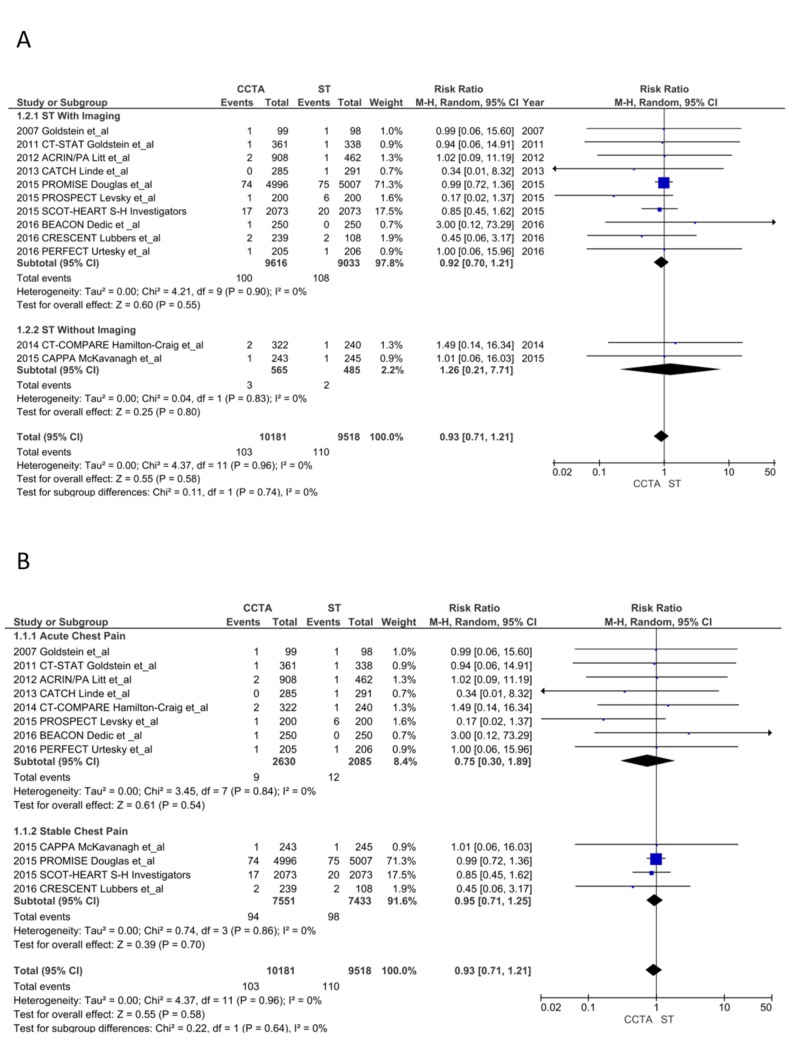
All-cause mortality CCTA, coronary computed tomography angiography; ST, stress testing. A. ST with imaging vs. ST without imaging  [13-28] B. Acute chest pain (ACP) vs. stable chest pain (SCP) [13-28]

All-cause mortality: There was no difference in all-cause mortality (103 vs. 110; RR = 0.93, CI = 0.71-1.21; P = .58, I^2^ = 0%). The subgroup analyses for ST with imaging (RR = 0.92, CI = 0.70-1.21; P = .55, I^2^ = 0%), ST without imaging (RR = 1.26, CI = 0.21-7.71; P = .80, I^2^ = 0%), ACP (RR = 0.75, CI = 0.30-1.89; P = .54, I^2^ = 0%) and SCP (RR = 0.95, CI = 0.71-1.25; P = .70, I^2^ = 0%) found no differences (Figure 3A and 3B) [13-28].

**Figure 4:**
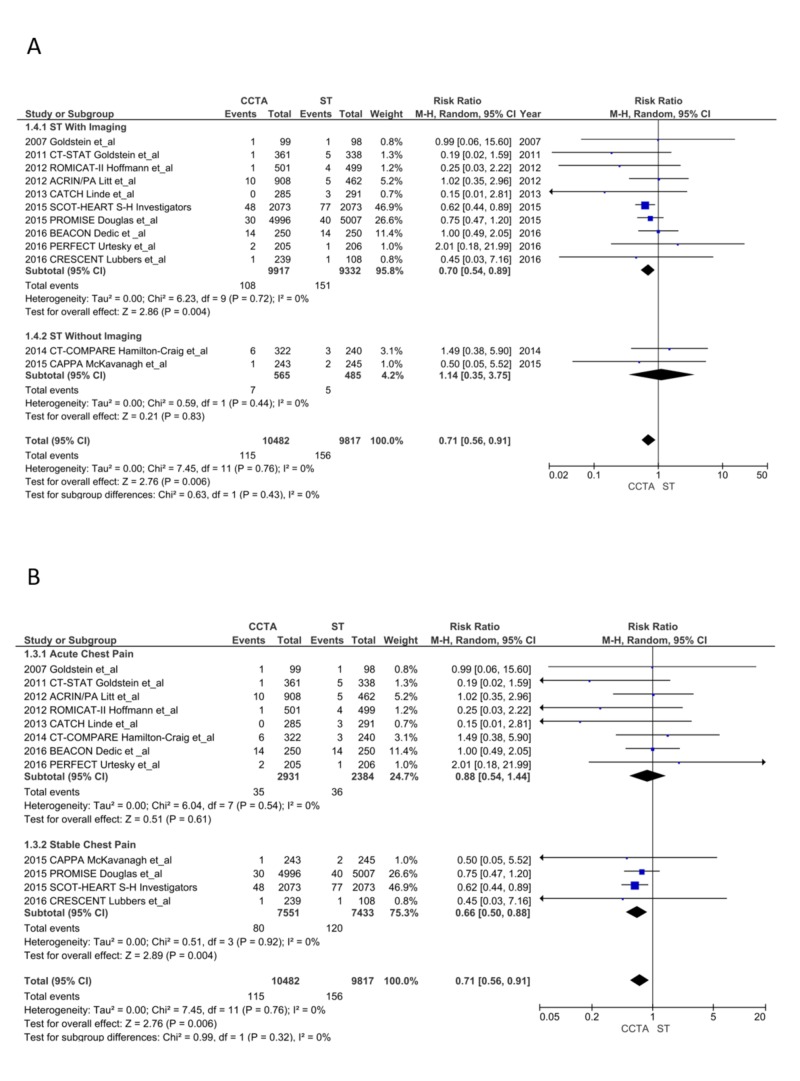
New myocardial infarction during the follow-up period CCTA, coronary computed tomography angiography; ST, stress testing; CI, confidence interval. A. ST with imaging vs. without imaging [13-28] B. Acute chest pain vs. stable chest pain [13-28]

New MI during follow-up period*:* A significant reduction in the incidence of future MI was noticed in the CCTA arm (115 vs. 156; RR = 0.71, CI = 0.56-0.91; P < .006, I^2^ = 0%); this was mainly noted as a reduction in MI in the SCP subgroup patients (80 vs.120; RR = 0.66, CI = 0.50-0.88; P = .004, I^2^ = 0%) compared to the ACP subgroup that showed no difference (35 vs. 36; RR = 0.88, CI = 0.54-1.44; P = .61, I^2^ = 0%). The CCTA arm also had significantly reduced MIs compared to ST with imaging (RR = 0.70, CI = 0.54-0.89; P = .004, I^2^ = 0%) with no difference compared to ST without imaging (RR = 1.14, CI = 0.35-3.75; P = .83, I^2^ = 0%; Figure 4A and 4B) [13-28]. The NNT after CCTA to prevent one MI was 204 and NNT after ICA to prevent one MI was nine.

Secondary Endpoints

**Figure 5:**
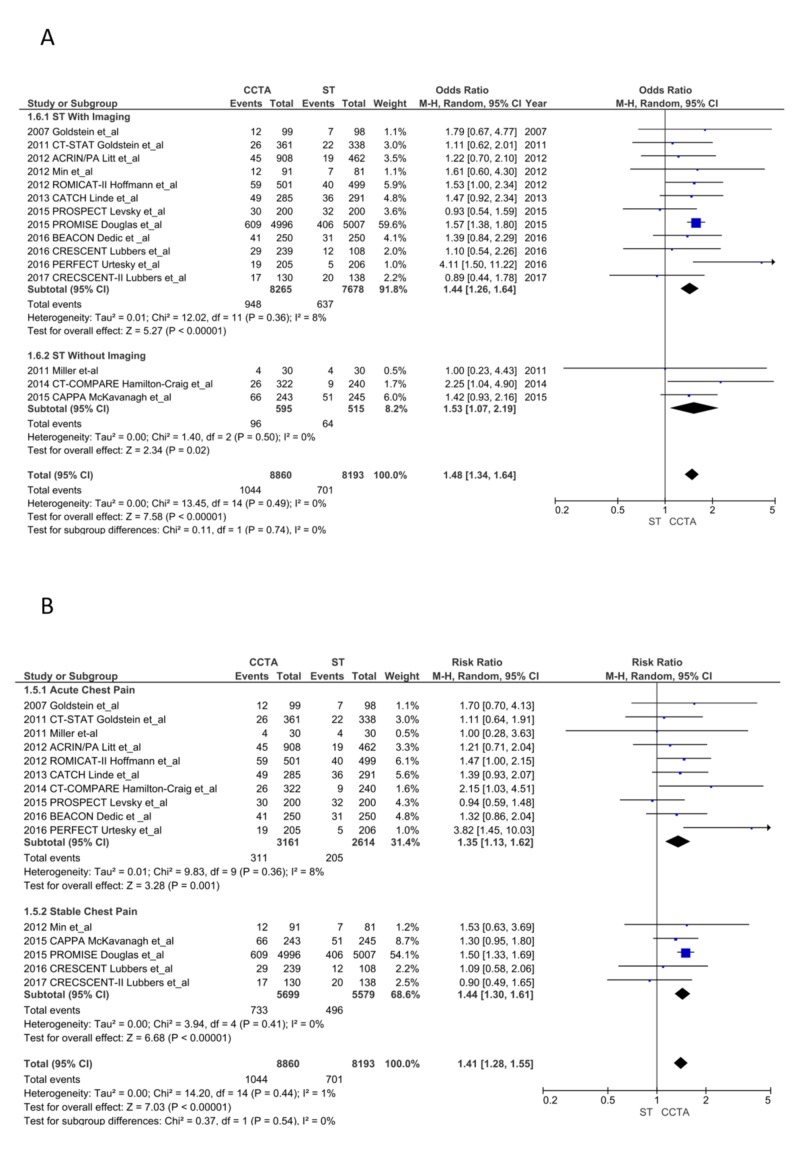
Invasive coronary angiograms CCTA, coronary computed tomography angiography; ST, stress testing. A. ST with imaging vs. without imaging [13-28] B. Acute chest pain vs. stable chest pain [13-28]

ICA after ST*: *The CCTA arm had significantly increased ICA (1,044 vs. 701; RR = 1.41, CI = 1.28-1.55; P < .00001, I^2^ = 1%). Both the ACP (311 vs. 205; RR = 1.35, CI = 1.13-1.62; P = .001, I^2^ = 8%) and SCP (733 vs. 496; RR = 1.44, CI = 1.30-1.61; P < .00001, I^2^ = 0%) subgroups had more ICA post-CCTA. ICA was common after CCTA compared to ST with imaging (RR = 1.37, CI = 1.21-1.55; P < .00001, I^2^ = 11%) and without imaging (RR = 1.37, CI = 1.21-1.55; P < .00001, I^2^ = 11%; Figure 5A and 5B) [13-28]. We did not include ICA from the SCOT-HEART study as they only reported new or canceled ICA in their manuscript and appendix [23].

**Figure 6:**
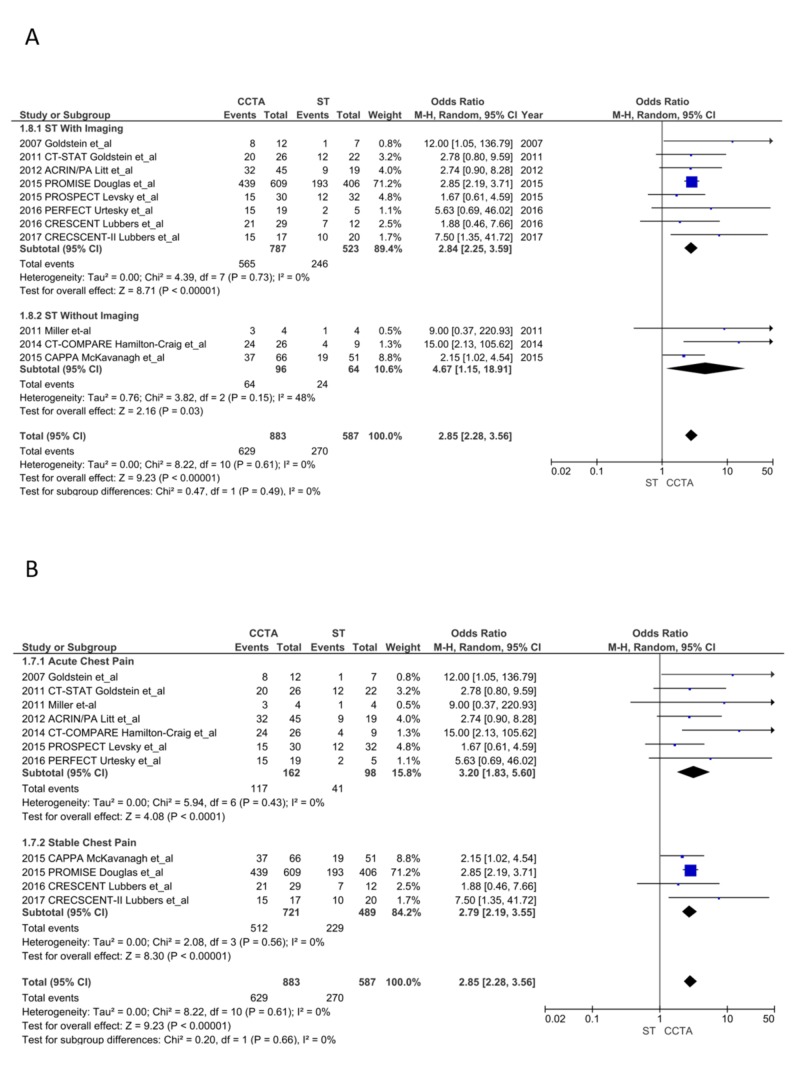
True positive invasive coronary angiograms CCTA, coronary computed tomography angiography; ST, stress testing. A. ST with imaging vs. without imaging [13-28] B. Acute chest pain vs. stable chest pain [13-28]

True positive ICA: CCTA lead to a significantly higher diagnosis of obstructive CAD (stenosis ≥ 50%) compared to ST (629/883 after CCTA vs. 270/587 after ST; OR = 2.85, CI = 2.28-3.56; P < 0.00001, I^2^ = 0%). This finding was consistent in both the ACP (OR = 3.20, CI = 1.83-5.60; P < .001, I^2^ = 0%) and SCP (OR = 2.79, CI = 2.19-3.55; P < .00001, I^2^ = 0%) subgroups and in ST with imaging (OR = 2.84, CI = 2.25-3.59; P < .00001, I^2^ = 0%) and without imaging (OR = 4.67, CI = 1.15-18.91; P = .03, I^2^ = 48%; Figure 6A and 6B) [13-28].

Revascularization: Revascularization (percutaneous coronary intervention and coronary artery bypass grafting) was significantly higher after CCTA (789 vs. 472; OR = 1.84, CI = 1.44-2.35; P < .00001, I^2^ = 53%). Of note, I^2^ was reduced to 0% with exclusion of the SCOT-HEART trial and without affecting significance [24]. This trend was consistent on subgroup analysis of ST with imaging (RR = 1.77, CI = 1.34-2.33; P < .00001, I^2^ = 60%), ST without imaging (RR = 2.36, CI = 1.40-3.98; P = .001, I^2^ = 0%), ACP (175 vs. 82; OR = 1.95, CI = 1.42-2.69; P < .0001, I^2^ = 17%), and SCP (614 vs. 390; OR = 1.70, CI = 1.16-2.51; P = .007, I^2^ = 77% and 0% without inclusion of the SCOT-HEART trial [23].

New unstable angina: There was no difference in new unstable anginas in the CCTA group vs. ST group (257 vs. 198; RR = 1.18, CI = 0.99-1.41; P = .06, I^2^ = 0%). A similar trend was seen on subgroup analysis of ACP (118 vs. 84; RR = 1.15, CI = 0.90-1.48; P = .27, I^2^ = 0%), SCP (139 vs. 114; RR = 1.21, CI = 0.93-1.58; P = .15, I^2^ = 4%), ST with imaging (RR = 1.18, CI = 0.98-1.40; P = .07, I^2^ = 0%) and ST without imaging, (RR = 1.09, CI = 0.20-5.92; P = .92, I^2^ = 49%).

**Figure 7:**
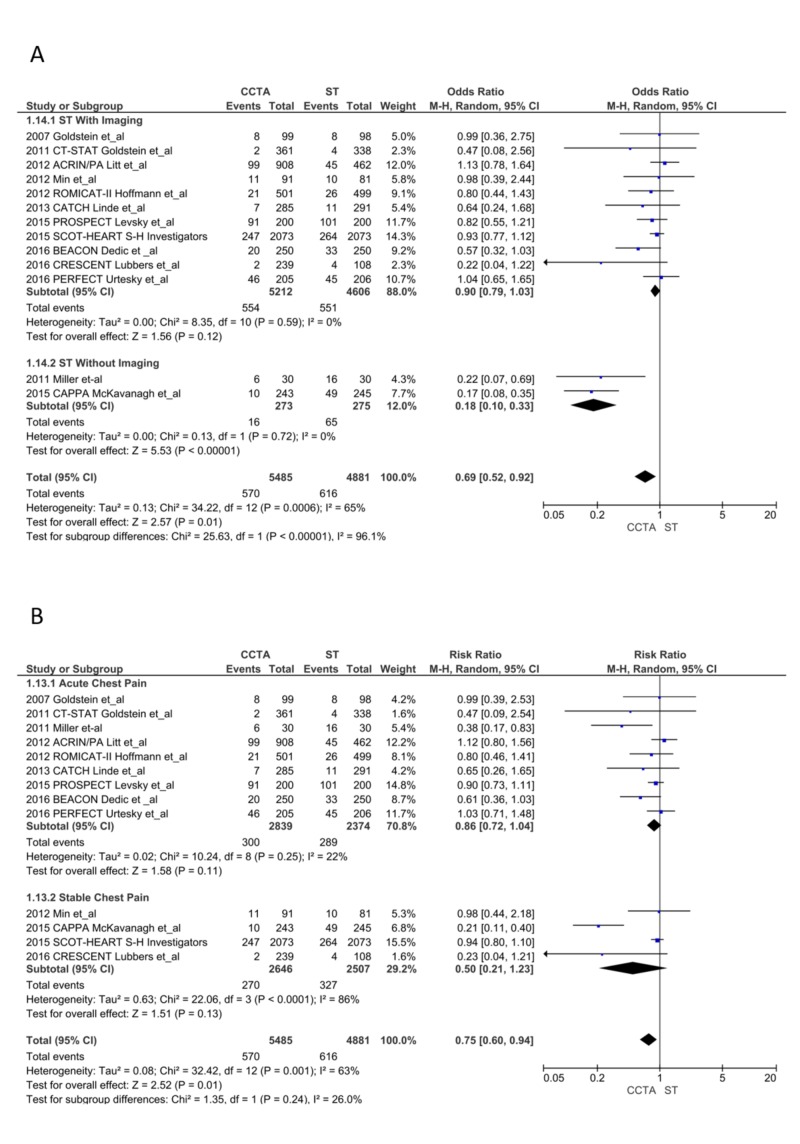
Emergency room visits or hospital admissions during the follow-up period CCTA, coronary computed tomography angiography; ST, stress testing. A. ST with imaging vs. without imaging [13-28] B. Acute chest pain vs. stable chest pain [13-28]

ER visits and/or hospital admissions during the follow-up period: ER visits and/or hospital admissions were reduced significantly in the CCTA arm (570 vs. 616; RR = 0.75, CI = 0.60-0.94; P = .01, I^2^ = 63%). I^2^ was reduced to 16% without the CAPPA trial, but the results became statistically insignificant. The subgroup analysis of ACP and SCP and ST with imaging revealed no difference between CCTA and ST, though there were significantly reduced ER visits or hospital admissions in the CCTA arm compared to ST without imaging (RR = 0.27, CI = 0.15-0.48; P < .0001; I^2^ = 27%; Figure 7A and 7B) [13-28].

**Figure 8:**
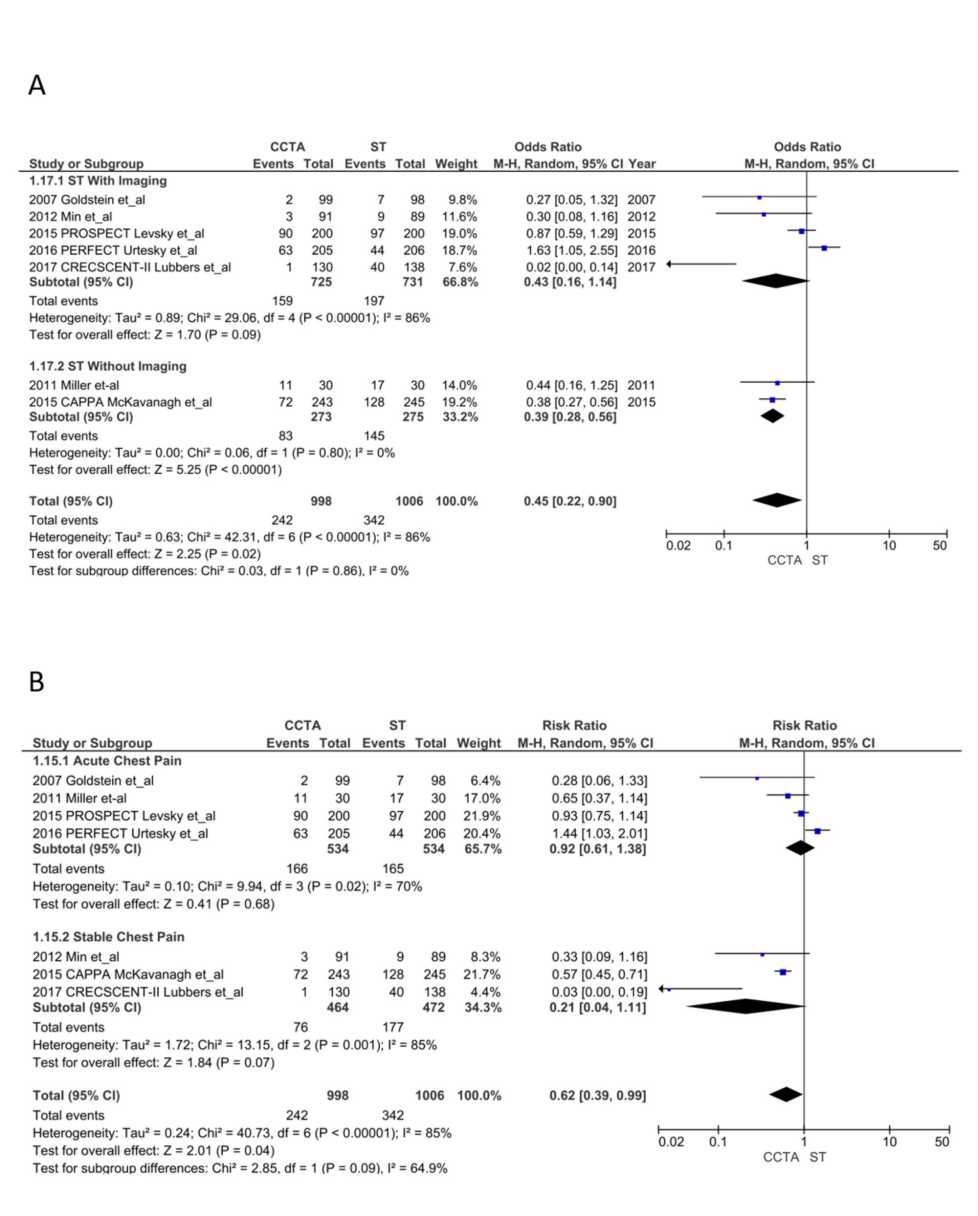
Follow-up tests CCTA, coronary computed tomography angiography; ST, stress testing. A. ST with imaging vs. without imaging [13-28] B. Acute chest pain vs. stable chest pain [13-28]

Follow-up tests: Patients in the CCTA arm had a significant reduction in downstream testing (242 vs. 342; OR = 0.45, CI = 0.22-0.90; P = .02, I^2 ^= 86%); sensitivity analysis did not reduce the heterogeneity. The ST with imaging subgroup (RR = 0.43, CI = 0.16-1.14; P = .09, I^2^ = 86%) and ACP subgroup (RR = 0.83, CI = 0.44-1.55; P = .56, I^2^ = 70%) showed no difference in follow-up testing. ST without imaging (RR = 0.39, CI = 0.28-0.56; P < .00001, I^2^ = 0%) and the SCP subgroup (RR = 0.17, CI = 0.04-0.77; P = .02, I^2^ = 80%) had a significant reduction in follow-up testing after CCTA (Figure 8A and 8B) [13-28].

Complications associated with CCTA vs. ST: Only four studies reported serious complications attributed to investigation modalities used in the trials. We did not identify any difference between the two arms (7 vs. 7; RR = 0.98, CI = 0.35-2.74; P = .96, I²=0%).

Direct discharge from the ER:* *Five studies reported direct ER discharges without admission to the hospital (CCTA arm = 936 vs. ST arm = 421; OR = 1.45, CI = 0.63-3.30; P = .38, I²=94%); sensitivity analysis did not reduce heterogeneity.

Cost analysis: Eight studies reported cost, but only five studies were usable as these reported mean cost and standard deviation.

Three studies reported the ER costs. There was a trend towards a decrease in ER costs in the CCTA arm (standardized mean difference [SMD] = -4.68, CI = -10.38 to 1.01; P = .11, I²=100%). Sensitivity analysis, without the CT-COMPARE trial, reduced the heterogeneity to 0% and the results became statistically significant (SMD = -0.38, CI = -0.51 to -0.26; P = .00001, I²=0%).

Five studies reported the total cost. There was no difference between the two arms (SMD = -0.64, CI = -1.75 to 0.46; P = .25, I²=99%). Sensitivity analysis without the CT-COMPARE reduced the heterogeneity to 45%; however, the results remained statistically insignificant. The subgroup analysis for the cost in the United States and cost elsewhere also had significant heterogeneity with no difference between the subgroups (chi-squared = 0.15, degrees of freedom = 1, P = .69, I²=0%).

Radiation dose: Four studies reported the cumulative radiation exposure usable for our analysis. The CCTA arm had significantly higher radiation exposure (SMD = 0.47, CI = 0.08-0.86; P = .02, I²=97%). Sensitivity analysis failed to reduce the heterogeneity.

## Discussion

Our meta-analysis of 21,210 patients comparing CCTA to ST demonstrated a significant reduction in the primary endpoint of MIs in the CCTA group without any difference in mortality. The reduction in MI was secondary to a significantly reduced number of events in the SCP group. The reduction in MIs is likely due to the early diagnosis of obstructive CAD and subsequent early initiation of aggressive medical management and revascularizations. Recently published five-year outcomes of the SCOT-HEART trial, which enrolled patients with SCP, also showed a significant reduction in MIs over five years [30]. This discrepancy in downstream MIs between the ACP and SCP group calls for a novel assessment strategy to risk-stratify ACP patients who present to the ER regarding invasive versus conservative management. The lack of mortality benefit in our analysis may not be evident because of the short follow-up times of the individual studies (four weeks to 25 months) compared to the five-year outcomes of the SCOT-HEART trial which showed a significant reduction in mortality from coronary heart disease or nonfatal MI than standard care alone [30].

This analysis also showed increased ICA and revascularizations, which also lead to significantly reduced MIs (NNT of nine to prevent one MI for each ICA). This early difference in ICA and revascularization may be lost after an extended follow-up as suggested by the five-year outcomes of the SCOT-HEART study [23]. This indicates that CCTA use leads to early diagnosis of CAD and subsequent early intervention compared to the ST, where patients eventually needed ICA and revascularization at the cost of increased MIs and mortality. Due to the high sensitivity of CCTA (approximately 99%), a negative CCTA may reduce further testing whereas a positive CCTA leads to additional invasive procedures. In our analysis, there were significantly more ICA, true positive ICA, and revascularizations, with significantly reduced follow-up tests. The use of CCTA leads to a higher number of invasive procedures, including revascularization, ultimately leading to higher costs overall. 

After the initial randomization and workup with either CCTA or ST, ER visits and rehospitalizations were significantly reduced in the CCTA arm; this differs from a previously published meta-analysis that showed no difference in ER visits and rehospitalizations [7-11]. A limitation of our analysis was the presence of substantial heterogeneity, making it difficult to generalize the results. The sensitivity analysis reduced heterogeneity with a trend towards reduced ER visits or rehospitalizations in the CCTA arm. Reduction in ER visits and rehospitalizations is promising, as earlier studies found that the reduced MIs after CCTA group was offset by increased future rehospitalizations and downstream costs.

The increased rates of angiographically confirmed CAD post-CCTA is another significant finding that suggests that CCTA has a better positive predictive value than ST (with or without imaging) to identify obstructive CAD at a time when current guidelines do not support the routine use of CCTA in intermediate-risk patients. Although our analysis showed an increasing trend towards unstable anginas in the CCTA arm, we hypothesize that this trend is likely the consequence of higher rates of revascularization in the CCTA group.

The cost analysis had substantial heterogeneity for both ER visits and downstream costs. The trials included in our analysis were conducted in different countries with different healthcare systems and cost structures [13-28]. In our analysis, even though a trend towards decreased ER costs was seen in the CCTA arm, there was no clear advantage of total downstream cost to either imaging strategy. In the absence of any significant mortality benefit, it is reassuring that whichever approach the provider offers will not adversely affect the patient. CCTA was associated with significantly higher cumulative radiation exposure; however, there was substantial heterogeneity, likely due to different scanners used in various trials.

Limitations

Our study had several significant limitations. First, a lack of long-term follow-up in the individual RCTs (≤25 months) that may not include events, hospitalizations, and revascularizations beyond 25 months would magnify the risks of ICA and revascularization and obscure potential long-term benefits. This may be true for ACP trials as short follow-up may have masked the advantage for either arm. Also, some outcomes were not reported by most studies, leading to substantial heterogeneity that persisted even after sensitivity analysis. In addition, we were unable to estimate radiation exposure from all studies between the two groups since they reported data in a variable form. Also, only three studies used ST without imaging, and the other studies used a combination of imaging and non-imaging ST; this leads to substantial overlap between the groups and has a risk to introduce bias in our results. Finally, these trials, although relatively modern, did not utilize high-sensitivity cardiac troponin tests. Their hypotheses must be tested again with the advent of these tests.

## Conclusions

Our analysis is the largest to date of 16 RCTs and found a significant reduction in post-CCTA MIs with increased ICA and revascularizations. In the future, more RCTs are needed utilizing scoring methods to identify more robust downstream investigations, cost analysis, and radiation exposure.
